# Inflammatory and Antioxidant Pattern Unbalance in “Clopidogrel-Resistant” Patients during Acute Coronary Syndrome

**DOI:** 10.1155/2015/710123

**Published:** 2015-03-19

**Authors:** Raffaele Caruso, Silvia Rocchiccioli, Anna Maria Gori, Antonella Cecchettini, Betti Giusti, Guido Parodi, Lorena Cozzi, Rossella Marcucci, Marina Parolini, Ilaria Romagnuolo, Lorenzo Citti, Rosanna Abbate, Oberdan Parodi

**Affiliations:** ^1^National Research Council, Institute of Clinical Physiology, Cardiothoracic and Vascular Department, Niguarda Ca' Granda Hospital, Piazza Ospedale Maggiore 3, 20162 Milan, Italy; ^2^National Research Council, Institute of Clinical Physiology, Via Moruzzi 1, 56124 Pisa, Italy; ^3^Department of Invasive Cardiology 1, Careggi Hospital, University of Florence, Largo Brambilla 3, 50134 Florence, Italy; ^4^Department of Invasive Cardiology 1, Careggi Hospital, Largo Brambilla 3, 50134 Florence, Italy; ^5^IRCCS, Fondazione Don Gnocchi, Via di Scandicci 269, 50143 Firenze, Italy; ^6^Department of Clinical and Experimental Medicine, University of Pisa, Via Roma 5, 56100 Pisa, Italy

## Abstract

*Background*. In acute coronary syndrome (ACS), inflammation and redox response are associated with increased residual platelet reactivity (RPR) on clopidogrel therapy. We investigated whether clopidogrel interaction affects platelet function and modulates factors related to inflammation and oxidation in ACS patients differently responding to clopidogrel. *Material and Methods*. Platelet aggregation was measured in 29 ACS patients on dual (aspirin/clopidogrel) antiplatelet therapy. Nonresponders (NR) were defined as RPR ≥70% by ADP. Several inflammatory and redox parameters were assayed and platelet proteome was determined. *Results*. Eight (28%) out of 29 ACS patients resulted NR to clopidogrel. At 24 hours, the levels of Th2-type cytokines IL-4, IFN*γ*, and MCP-1 were higher in NR, while blood GSH (r-GSH_bl_) levels were lower in NR than responders (R). Proteomic analysis evidenced an upregulated level of platelet adhesion molecule, CD226, and a downregulation of the antioxidant peroxiredoxin-4. In R patients the proinflammatory cytokine IL-6 decreased, while the anti-inflammatory cytokine IL-1Ra increased. *Conclusions*. In patients with high RPR on clopidogrel therapy, an unbalance of inflammatory factors, platelet adhesion molecules, and circulatory and platelet antioxidant molecules was observed during the acute phase. Proinflammatory milieu persists in nonresponders for a long time after the acute event while antioxidant blood factors tend to conform to normal responsiveness.

## 1. Introduction

Since platelet activation plays a critical point in the pathogenesis of ACS [1], antiplatelet treatments are frequently used in patients with acute coronary syndrome (ACS), in order to reduce ischemic events. The dual antiplatelet therapy that combines aspirin (acetylsalicylic acid, ASA) and a platelet P2Y_12_ receptor inhibitor is the standard antithrombotic strategy in the treatment of ACS and for the prevention of thrombotic complications subsequent to percutaneous coronary intervention (PCI) [2]. Despite the availability of newer platelet P2Y_12_ receptor inhibitors, clopidogrel remains widely used, also because it allows a considerable cost-containment. However, notwithstanding significant benefits reported with dual antiplatelet treatment in major clinical trials, the occurrence of major ischemic events, including stent thrombosis, remains a serious clinical problem [3]. Several studies have revealed variability of antiplatelet efficacy based on the so-called “clopidogrel resistance” [4–6]. Lack of adequate platelet inhibition to clopidogrel might be associated with many factors including patient noncompliance, drug interactions, alternative mechanisms of platelet activation, metabolic pathway alterations, and also polymorphisms of genes that play a role in clopidogrel metabolism [7, 8].

Inflammation plays an important role in the pathophysiology and prognosis of ACS. The extent of inflammatory response is crucial in haemostatic system, throughout increased platelet reactivity and endothelial dysfunction, factors which are potentially involved in the variability of antiplatelet efficacy [9]. In this context, elevated leukocyte count and high levels of anti-inflammatory cytokine interleukin- (IL-) 10 are reported as important factors associated with the probability of low clopidogrel responsiveness [10]. Differently, an inverse relationship between anti-inflammatory cytokine IL-4 and the residual platelet reactivity was reported in ACS patients on dual antiplatelet therapy [11], suggesting a complex interaction between inflammatory pathways and response to clopidogrel.

Experimental and clinical studies suggest also a pivotal role of reactive oxidant species (ROS) in the mechanism of platelet activation [12]. ROS are implicated in platelet activation by inactivating nitric oxide (NO) and releasing platelet agonists and proatherogenic molecules [12]. In particular, the main antioxidant amino-thiol glutathione (GSH) has a central role in the redox modulation of platelet activity [13], and it is known that deficiencies of enzymes that are involved in GSH synthesis are associated with cardiovascular events [14]. Moreover, in human platelets, NO pathway is a target of the prooxidant amino-thiol homocysteine (Hcy), which promotes the reduction of NO, platelet hyperactivation, and other thrombogenic processes [15, 16].

More recently, with the omics booming, proteomics approaches have been often adopted to investigate platelet proteome. In a previous proteomics study it was reported that ASA-resistant patients show an increased plasma expression of vitamin D-binding protein isotypes [17]. This was associated with the failure of ASA to prevent both thromboxane A2 production and platelet activation, unmasking a potential mechanism involved in ASA resistance. Moreover, platelet protein profiling of patients with stable angina under clopidogrel therapy shows different expression of proteins involved in the cytoskeleton rearrangement, in the energetic metabolism, and in the oxidative stress [18].

The aim of this study was to associate several soluble circulating inflammatory markers and redox factors in ACS patients with different responses to clopidogrel treatment. In a restricted subgroup of patients [6], mass spectrometry-based protein profile of platelet proteome was investigated and information on oxidative, inflammatory pathways and energetic metabolism was obtained.

## 2. Material and Methods

### 2.1. Population Enrolment and Platelet Reactivity Assessment

Twenty-nine ACS patients undergoing PCI, stent implantation in dual antiplatelet therapy with clopidogrel, and ASA were enrolled at the University Hospital of Careggi in Florence. Platelet reactivity assessment was made by light transmittance aggregometry (APACT4, Helena Laboratories, Milan, Italy) using adenosine diphosphate (ADP) as an agonist [19]. Blood samples anticoagulated with 0.109 M sodium citrate (ratio, 9 : 1) were obtained 24 hours, 1 week, and 1 month after a 600 mg clopidogrel loading dose. Platelet-rich plasma, obtained by centrifuging whole blood for 10 minutes at 200 g, was stimulated with 10 *μ*M ADP. The 100% line was set using platelet-poor plasma and the 0 baseline established with platelet-rich plasma (adjusted from 180 × 109/L to 300 × 109/L). Platelet aggregation (according to the Born method) was evaluated considering the maximal percentage of platelet aggregation in response to stimulus. Runs of 10 minutes were set for all the agonists used in the LTA test in order to better characterize the profile of aggregation/disaggregation. Maximum aggregation was used in order to measure the entity of platelet inhibition. The coefficient of variation of ADP platelet aggregation was 6.8%. Platelet reactivity was evaluated within 24 hours from clopidogrel loading. High residual platelet reactivity by ADP test was defined as platelet aggregation of 70% or greater [20].

The study complied with the principles of the Declaration of Helsinki, and Careggi Hospital Ethics Committee approved the study protocol. All the patients gave written informed consent to participate in the study. Patients with poor adherence to the prescribed therapy were excluded from the study.

### 2.2. Assessment of Inflammatory Parameters

In all enrolled ACS patients, blood (Na^2+^-EDTA and 4% trisodium citrate as anticoagulants) samples were collected at 24 hours, 1 week, and 1 month from administration of dual antiplatelet therapy for the assessment of a panel of inflammatory mediators, consisting of cytokines associated with Th1/Th2-type T cells (interleukin- (IL-) 4, IL-12, and interferon-gamma (IFN-*γ*)), pro- (IL-6 and tumor necrosis factor alpha (TNF-*α*)), and anti-inflammatory cytokines (IL-10 and receptor antagonist of IL-1 (IL-1ra)), and chemotactic molecules (interferon-gamma-induced protein 10 (IP-10 or CXCL10), interferon-inducible T cell alpha chemoattractant (I-TAC or CXCL11), monocyte chemotactic protein-1 (MCP-1), and macrophage inflammatory protein-1*β* (MIP-1*β* or CCL4)).

Plasma inflammatory mediators were determined by Bio-Plex cytokine assay (R&D Systems, Minneapolis, MN, USA) according to manufacturer's instructions.

### 2.3. Assessment of Redox Status

In 23 out of 29 enrolled ACS patients, additional blood samples were collected at 24 hours, 1 week, and 1 month after administration of dual antiplatelet therapy for the assessment also of reduced and total aminothiols, Hcy, and GSH, and the activities of GSH peroxidase type-1 (GPx-1). Blood reduced GSH (r-GSH_bl_) level was determined by prompt acidification of whole blood immediately after blood sample collection according to method previously described [14]. As reduced GSH levels in plasma are low (1-2%), the reduced GSH concentration in whole blood can reflect GSH content inside the cellular fraction of blood. Plasma reduced and total forms of Hcy and blood total GSH were determined according to methods validated in our laboratory [14]. The total form of GSH measured in our study includes the oxidized GSH, all conjugated forms of GSH (protein-bound GSH and GSH-mixed disulfides), produced through oxidative processes or thiol-disulfide exchange reactions, and reduced free GSH. Thiol separation was performed by high-performance liquid chromatography (HPLC) method (ProStar-Varian, Surrey, UK). Erythrocyte GPx-1 was determined using t-butyl hydroperoxide (SIGMA, Steinheim, Germany) as substrate [21]. GPx-1 activities were determined in diluted purified red blood cells, stored frozen up to analysis which was performed within 1 month from storage.

### 2.4. Platelet Isolation, Protein Extraction, Reduction, Alkylation, and Digestion

In a restricted population of 12 ACS patients (6 nonresponders), platelet-rich plasma (PRP) was obtained from whole blood at 24 hours after administration of dual antiplatelet therapy. PRP was prepared by centrifuging blood samples at 150 g for 10 minutes. Then it was removed and platelet-poor plasma was prepared by further centrifugation at 3,000 g for 3 minutes. PRP was adjusted with autologous platelet-free plasma to reach a platelet count between 180000 plt/*μ*L and 300,000 plt/*μ*L. The purity of isolated platelets was confirmed by flow cytometry on a Becton Dickinson FACS can instrument.

**Figure 1:**
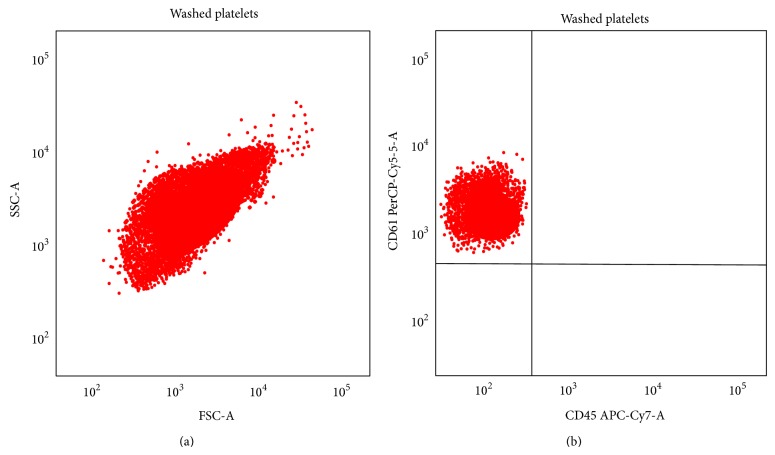
Flow cytometric determination of white blood cell (WBC) contamination of platelet suspensions. Flow cytometric analysis after staining with antibodies specific for CD61 (PerCP-Cy5-5) and for CD45 (APC-Cy7). In (a), the box includes cellular suspension according to SSC and FSC. In (b) the CD61+/CD45+ events gated on P1 are shown in the right upper quadrant.

White blood cells in platelet suspension were identified through their expression of CD45. A volume of 10 *μ*L of platelet suspension was incubated for 20 min in the dark with (1) peridinin chlorophyll protein-cyanin-5.5- (PerCP-Cy5.5-) labelled monoclonal antibodies against human CD61 (Becton Dickinson, San Jose, USA); (2) allophycocyanin-cyanin-7- (APC-Cy7-) labelled monoclonal antibodies against human CD45 (Becton Dickinson, San Jose, USA) (Figure 1).

PRP samples were centrifuged at 800 g for 15 min and pellets of platelets were resuspended in 350 *μ*L of lysis solution (Tris/HCl 50 mM, 0.1% Triton-X100). Detergent was removed using detergent removal columns (Pierce) following manufacturer instructions. Briefly, protein extracts were placed on the column and incubated for two minutes. Columns were centrifuged for two minutes to collect detergent-free samples. BCA assay was used to evaluate total protein concentration. 100 *μ*L of protein extract (1 *μ*g/*μ*L concentration) was added to 100 *μ*L of 40 mM of ammonium hydrogen carbonate (pH = 8). Reduction was obtained by adding 1 *μ*L of 1 M dithiothreitol to each sample, with an incubation of 20 min at 80°C. For alkylation, 20 *μ*L of 100 mM iodoacetamide was added to the samples and incubated for 30 min at 37°C. Trypsin digestion was performed incubating samples with 8 microL of 0.25 mg/ml solution at 37°C overnight.

### 2.5. LC-MS/MS Analysis

Chromatographic separation of peptides was performed using an ultimate 3000 nano-HPLC system (LC Packings, DIONEX, USA). 100 *μ*L of sample was added to a solution composed by 2% CH_3_CN and 0.1% formic acid up to 200 *μ*L of final volume. The loading pump preconcentrated the sample in a precolumn cartridge (PepMap-100 C18 5 mm 100 A, 30 mm id × 5 mm). Chromatographic separation of peptides was performed using a C18 PepMap-100 column (15 cm × 75 mm id, LC Packings DIONEX) equilibrated at 45°C with solvent A (water/acetonitrile 98/2 vol/vol, 0.1% formic acid) at a flow rate of 300 nL min^−1^. Runs were performed under 40 min linear gradient from 10 to 45% of solvent B (water/CH_3_CN 2/98 vol/vol, 0.1% formic acid) followed by 10 min of a purge step at 95% of B before a 20 min reequilibration step to the starting conditions. The column was directly coupled to TripleTOF 5600 System (AB SCIEX, Toronto, Canada), equipped with a DuoSpray ion source (AB SCIEX, Toronto, Canada). Eluted peptides were directly processed using TripleTOF 5600 mass spectrometer (AB SCIEX, Toronto, Canada). The mass spectrometer was controlled by Analyst 1.6.1 software (AB SCIEX, Toronto, Canada). For positive ionization, ion source parameters were the following: spray voltage 3 KV, temperature 150°C, curtain gas 25 psi, GS1 10 psi. For information dependent acquisition (IDA) analysis, survey scans were acquired in 250 ms. Dynamic exclusion was set for 1/2 of peak width (~8 s), and then the precursor was refreshed off the exclusion list.

### 2.6. Protein Identification and Label-Free Comparative Analysis

MS/MS data were processed with ProteinPilot Software (AB SCIEX, Toronto, Canada), using the Paragon and Pro Group Algorithms and SwissProt 2013 as protein database for* Homo sapiens* species. The false discovery rate analysis was done using the integrated tools in ProteinPilot software and a confidence level of 95% was set. The statistical comparative analysis was performed using MarkerView Software 1.2.1 (AB SCIEX). The ion chromatograms of high confidence peptides identified by ProteinPilot were extracted using PeakView Software and then MS peak areas and identifications were imported into MarkerView Software.

### 2.7. Statistical Analysis

Continuous variables were presented as median and interquartile ranges and categorical data as frequency (%). Group comparisons (responders versus nonresponders) of the clinical and biochemical characterizations were performed by unpaired Student's *t*-test for continuous variables or Mann-Whitney *U* test for not normally distributed variables, and Chi-square or Fisher's exact test for categorical variables. Time-dependent changes (24 hours, 1 week, and 1 month) in groups were assessed by nonparametric Friedman test (*P* for time); pairwise post hoc comparisons were performed by using the Wilcoxon signed-rank test with Bonferroni correction.

Pearson's correlation coefficient (*r*) and the equivalent nonparametric Spearman's rho (rho) correlation coefficient were used to analyze the association between biochemical variables.

For mass spectrometric data, principal component analysis (PCA) was performed with MarkerView 1.2 software in order to evidence groupings among the data set. All profile areas were normalized using total area sum. The two groups (responders (R) and nonresponders (NR)) were compared with *t*-test with a threshold of 95% (*P* value = 0.05) and fold change > 2. A *P* value <0.05 was considered statistically significant.

## 3. Results

### 3.1. Clinical Characteristics of Clopidogrel Nonresponder and Responder Patients

The median age of enrolled ACS patients was 75 (71–98) years. For sixteen (55%) patients ST-segment elevation myocardial infarction was characterized by electrocardiography, and 8 (28%) patients showed three-vessel coronary artery disease. Eight (28%) out of 29 ACS patients were considered NR to clopidogrel. The clinical characteristics of R and NR patients are summarised in Table 1.

The main clinical features of R and NR patients were similar, with exception of bare metal coronary stents and diabetes that were more frequent in NR patients. The medical history was comparable too, as well as therapy (in particular the administration of GPIIb/IIIa inhibitors) associated with clopidogrel-based antiplatelet treatment (Table 1).

### 3.2. Inflammatory and Redox Patterns in Clopidogrel Nonresponder and Responder Patients during Early Phases of Dual Antiplatelet Treatment

The inflammatory data concerning the acute phase of ACS at 24 hours after clopidogrel loading dose are reported in Table 2.

NR patients showed higher levels of IL-4 and IFN*γ* that are both associated with the differentiation of naive T cell Th1 and Th2. The levels of anti-inflammatory cytokine IL-10 were higher in NR than in R, while the levels of proinflammatory cytokines, IL-6 and TNF *α*, were comparable between the two groups. Among chemokines, MIP-1*β* was more concentrated in NR platelets, while I-TAC was present in higher levels in R patients.

Among inflammatory variables, in all patients, IL-4 levels were positively related only with MIP-1*β* levels (rho = 0.56, *P* = 0.008), while, in NR patients, MIP-1*β* levels were related with IL-10 levels (rho = 0.93, *P* = 0.001).

The redox data associated with clopidogrel resistance during the acute phase of ACS at 24 hours after clopidogrel loading dose are summarized in Table 3.

The levels of aminothiols, in both plasma and whole blood, were equivalent in the two patient groups, with the exception of the r-GSH_bl_, which was significantly lower in NR and positively related with plasma total GSH levels (rho = 0.51, *P* = 0.014). GPx-1 activities were also comparable between groups.

Among all patients, IL-4 levels were negatively related to plasma reduced GSH (rho = −0.59, *P* = 0.004) and TNF-*α* levels were negatively associated with r-GSH_bl_ (rho = −0.52, *P* = 0.016). Differently, I-TAC levels were positively correlated to plasma reduced GSH (rho = 0.56, *P* = 0.008).

### 3.3. Inflammatory and Redox Profiles in Responders and Nonresponders

**Figure 2:**
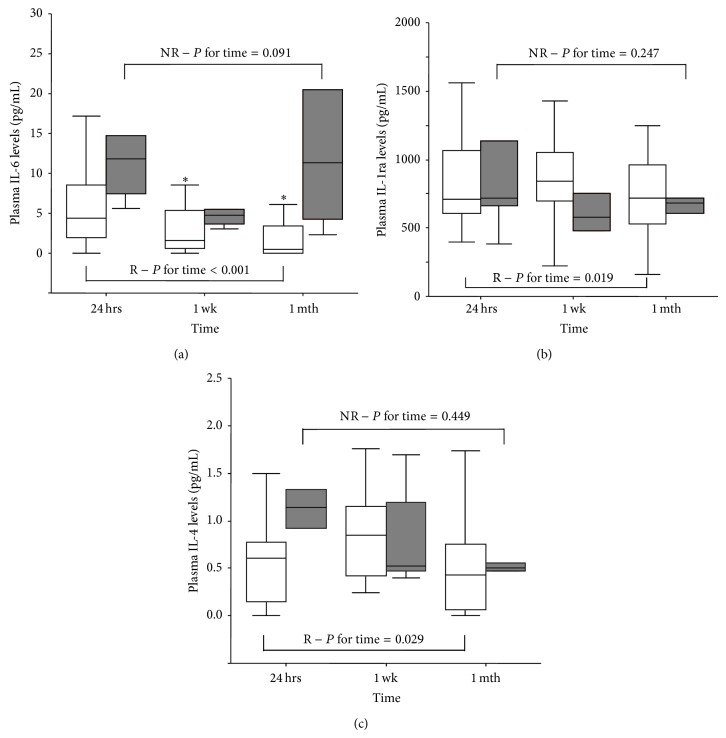
Profile of levels of IL-6 (a), IL-1ra (b), and IL-4 (c) in NR and R patients (R: empty box-plots; NR: dark box-plots). *P* for time refers to time-dependent changes (24 hours, 1 week, and 1 month) in groups assessed by nonparametric Friedman test, ^*^ *P* < 0.025 versus 24 hrs by Bonferroni adjusted post hoc test for pairwise comparisons.

Follow-up evaluation of inflammatory and redox profiles was assessed in 19 ACS patients (24 hours, one week, and one month after clopidogrel loading dose). In NR, the levels of inflammatory parameters were unchanged during the first month after dual antiplatelet therapy administration, with the exception of IL-6 that tended to decrease after one week from the acute phase. At variance, R patients showed significant changes of IL-6, IL-1ra, and IL-4 levels over time (Figure 2).

In particular, a reduction of IL-6 levels was observed in R patients (Figure 2(a)) resulting lower one month after treatment than in NR patients [0.6 (0.0–6.1) and 11.3 (3.3–54.6) pg/mL of IL-6 in R and NR patients, respectively *P* = 0.059]. Differently, an increment of the IL-1ra levels was observed at one week in R with respect to NR patients (Figure 2(b)). In addition, responders showed a significant increase of IL-4 levels one week after treatment, returning to baseline values at one month (Figure 2(c)). The levels of chemotactic molecules did not change over time in both groups with respect to those observed in the acute phase (Table 4).

**Figure 3:**
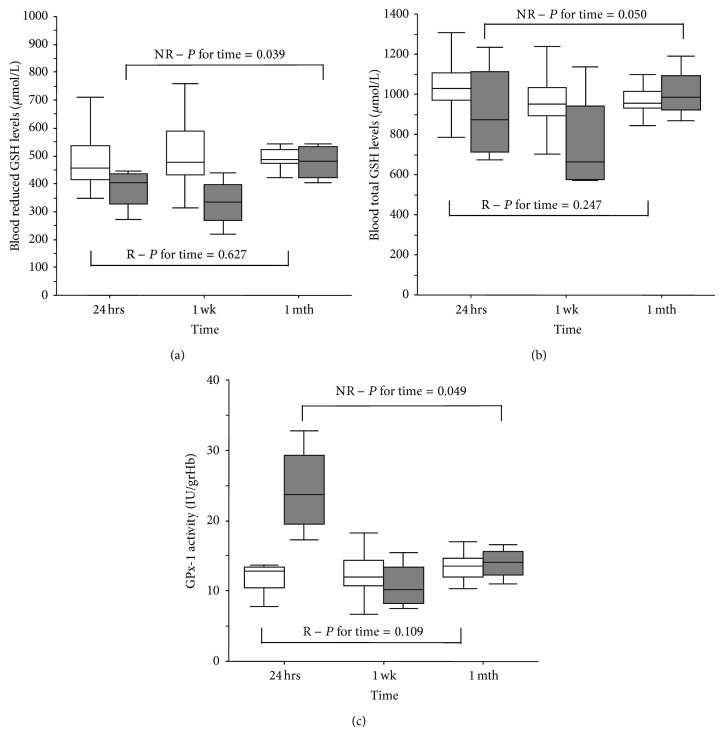
Profile of levels of blood reduced GSH (a), blood total GSH (b), and GPx-1 activities (c) in NR and R patients (R: empty box-plots; NR: dark box-plots). *P* for time refers to time-dependent changes (24 hours, 1 week, and 1 month) in groups assessed by nonparametric Friedman test.

Among redox variables, NR patients showed significant changes with respect to R patients of the r-GSH_bl_ levels, which tended to reequilibrate after one month (Figure 3(a)). Likewise, the levels of blood total GSH (t-GSH_bl_) were reduced one week after dual antiplatelet therapy administration (Figure 3(b)). In NR, the activity of GPx-1 reached an activity comparable to that of R patients within one week (Figure 3(c)). The levels of Hcy did not change over time in both groups as compared to the acute phase (Table 4).

### 3.4. Proteomics Analysis of Clopidogrel Responders and Nonresponders

**Figure 4:**
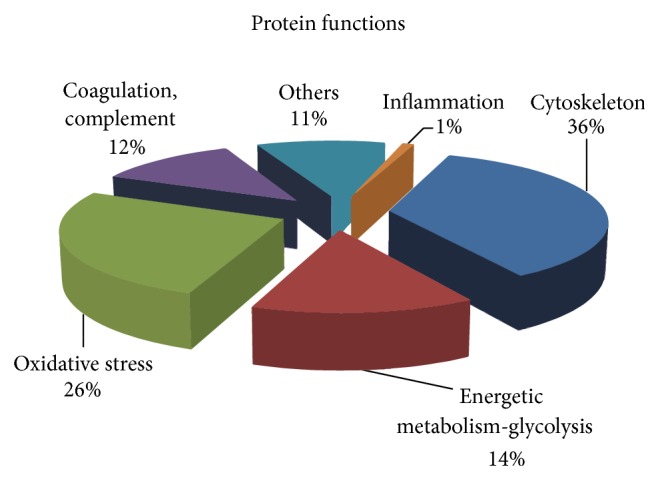
Pie charts of the 74 identified proteins. Proteins are divided on the basis of their functions based on Gene Ontology database (http://www.geneontology.org/).

The platelet proteome of 12 ACS patients (6 R and 6 NR) was analyzed by LC-MSMS approach 24 hours after clopidogrel loading dose. Individual profiles were obtained in 1 hour of chromatographic separation coupled with electrospray mass spectrometry. 74 proteins related to inflammation, oxidative state, and energetic metabolism were identified with a Protein Score (Confidence) > 95% and using local false discovery rate analysis >1% as stringent criterion to avoid false positives (Figure 4).

**Figure 5:**
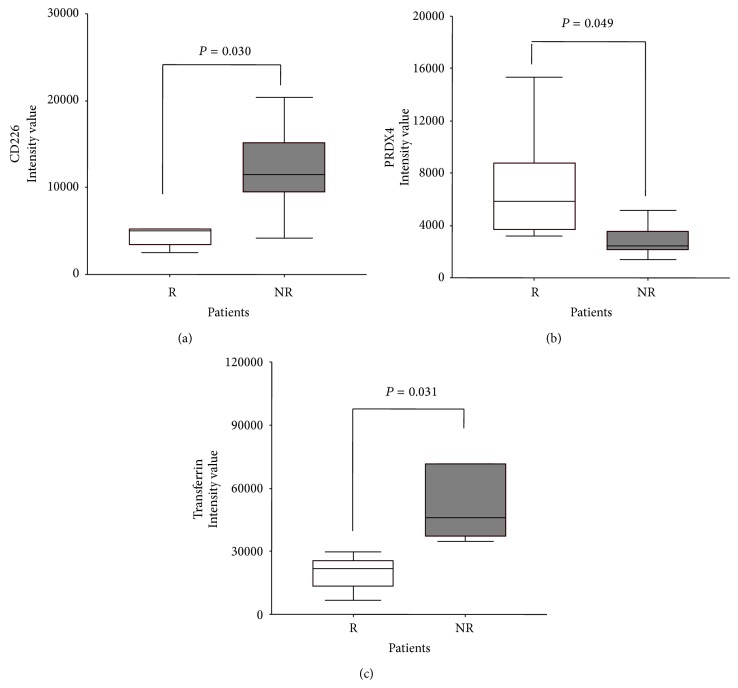
Platelet protein expression of CD226 (a), PRDX4 (b), and transferrin (c) in NR and R patients (R: empty box-plots; NR: dark box-plots).

Differentially expressed markers were evaluated using MarkerView software, and 3 proteins (CD226, peroxiredoxin-4 (PRDX4), and transferrin (TRFE)) were differently modulated between R and NR patients (Figures 5(a)–5(c)).

## 4. Discussion

In the present study, changes in expression and modulation of inflammation, redox, and platelet molecules were investigated in R and NR to clopidogrel-based antiplatelet treatment patients during ACS. In the acute phase of the coronary syndrome, the IL-4 and MIP-1*β* levels were found higher in NR than in R patients. IL-4, a known Th2-type anti-inflammatory cytokine, is crucial to modulate T cell differentiation and to direct differentiation of human monocytes into antigen presenting specialized cells [21]. The T cell differentiation into Th1 or Th2 is important in the clinical context of ACS since Th1 cells are able to activate monocytes/macrophages, committing them to the so-called “delayed-type hypersensitivity” phenomenon [22], and to induce the expression of factors promoting microthrombosis. Gori et al. [11] reported an inverse relationship between levels of IL-4 and the residual platelet reactivity in ACS patients undergoing PCI on dual antiplatelet therapy. In our study, NR patients showed higher levels of peripheral IL-4, data which seem to exclude a T cell differentiation towards the Th1 profile.

MIP-1*β* is known as CCR5 macrophage receptor ligand, involved in the activating signals for the monocyte recruitment during atherogenesis and in inflammatory processes [23]. Currently, the involvement of MIP-1*β* and CCR5 signals in response to antiplatelet therapy in ACS patients is poorly understood. In our study, the circulating levels of MIP-1*β* were found higher in NR than in R patients suggesting a greater tendency for the recruitment of monocytes. Likewise, few data are available regarding chemokine (C-X-C motif) receptor 3 (CXCR3) in response to antiplatelet therapy in ACS patients. CXCR3 receptor, preferentially expressed on activated Th1-cell population, is known to be involved in the recruitment of circulating activated immune cells [24]. CXCR3 receptor is activated by IFN-*γ*-inducible chemokines such as CXCL9, CXCL10 (IP-10), and CXCL11 (I-TAC), which are highly expressed in atherosclerotic lesions and play an important role in Th1-cell homing. Gori et al. reported higher levels of IP-10 in ACS patients on dual antiplatelet therapy with residual platelet reactivity, characterized also by elevated levels of IFN-*γ* with respect to R patients [11]. In our NR patients, despite confirmed higher levels of IFN-*γ*, circulating levels of I-TAC were lower. Altogether, these data support an involvement of CXCR3-binding chemokines in response to clopidogrel-based therapy in ACS patients, even if these data are not conclusive.

Of note, the levels of proinflammatory cytokine IL-6 decreased significantly only in R patients over time while the levels of anti-inflammatory cytokine IL-1ra are maintained elevated. These results suggest the presence of a more balanced inflammatory milieu in R with respect to NR patients; this impression is furthermore reinforced by elevated levels of IL-6 and MIP-1*β*, also persisting after one month from acute event.

In our study, the analysis of redox pattern has evidenced significantly lower levels of r-GSH_bl_ in the acute phase of NR compared to R patients. These data support the hypothesis that NR patients, during the acute phase, also present GSH depletion in addition to altered IL-4 and MIP-1*β* levels. Several studies suggest a critical role of ROS and of low molecular weight thiols, such as GSH, in modulation of platelet activation [25, 26]. In particular, in type-2 diabetes, the platelet hyperreactivity appears to be due to both deficiency of intraplatelet antioxidant status and reduced bioavailability of antithrombotic NO [27]. In ACS patients, the GSH-defective redox milieu might reflect a condition of oxidative stress or an increased production of ROS contributing to residual platelet reactivity. These findings are consistent with recent data by Becatti et al. [28] showing that ROS production is strictly correlated to the platelet aggregation and can be predictor of poor responsiveness to dual antiplatelet treatment. Moreover, the inverse correlations between the levels of GSH_bl_ and inflammatory parameters, such as IL-4 and I-TAC levels, suggest that the redox pattern, GSH-defective, might also affect the inflammatory pathway in ACS patients. Unlike the inflammatory state, the redox status in NR varies over time during therapy reaching the levels of the R patients (in particular the blood reduced GSH level and GPx-1 activity). This phenomenon might reflect a persistent action of the antioxidant system against oxidative stress. Nevertheless, the persistence of a greater inflammation in NR patients over time during antiplatelet therapy supports a more crucial role of the inflammation than redox state in response to clopidogrel.

Even analyzing the protein profile of platelets in a limited cohort of patients, the differential modulation of specific inflammatory and redox signals, in platelets of patients presenting different response to antiplatelet therapy, is confirmed and other involved markers are evidenced. CD226, a platelet adhesion molecule, is found overexpressed in platelet proteome of R patients [29]. In NR patients, the upregulation of platelet CD226 and the elevated levels of plasma MIP-1*β* highlight the presence of inflammatory mechanisms that promote adhesion of platelets and monocytes to vascular endothelial cells. Otherwise, the levels of PRDX4, an antioxidant enzyme crucial for the detoxification of hydrogen peroxide and superoxide, are reduced in NR patients. Accordingly, decreased platelet antioxidant content is known to be related to increased platelet aggregation [30]. TRFE minimizes the ability of circulating free iron to catalyze formation of free radicals. Indeed, TRFE levels are found reduced in patients with acute ischemic stroke, in conjunction with reduced values of the antioxidants GSH and GPx [31, 32]; furthermore, also thiols can reduce TRFE generating an increase of oxygen-derived free radicals [33]. Additionally, in presence of TRFE, a significant increase in lipid and protein oxidation and a decrease in GSH levels were observed in platelets [34]. Therefore, the different expression of PRDX4 and TRFE between R and NR and the low levels of blood GSH in NR highlight the role of redox-sensitive signals on platelet and the importance that these molecules could have in the effectiveness of clopidogrel-based therapy.

### 4.1. Study Limitations

A major limitation of this study is the restricted number of investigated patients. However, the simultaneous evaluation of inflammatory mediators and oxidative stress molecules in the bloodstream, over time during therapy, associated with the analysis of the platelet protein profile of ACS patients who differently respond to clopidogrel, makes it possible to provide an overview of the main modulated players in high RPR on clopidogrel therapy. Moreover, aspirin resistance and the relationship with high RPR on clopidogrel were not assessed and need further investigation.

## 5. Conclusions

The upregulation of circulating and platelet adhesion molecules and the presence of a defective platelet antioxidant system suggest that oxidative stress and inflammatory signals might favor platelet hyperactivity, which may be associated with clopidogrel response variability. Proinflammatory milieu persists in NR patients even at a late stage after the acute event, while the antioxidant molecules tend to conform to that of patients with normal responsiveness. An unbalance of inflammation and oxidation markers is not unexpected in the acute phase but follow-up evaluation evidences differential time-course between responders and nonresponders. A phenotypic fingerprint associated with high RPR in clopidogrel treatment is evidenced suggesting additional factors that might be potentially exploited as clinically useful predictors of resistance-related poststent complications.

## Figures and Tables

**Table 1:** Clinical characteristics between clopidogrel-responder and -nonresponder patients.

	Responders (*n* = 21)	Nonresponders (*n* = 8)	*P*
Age, years	73 (60–80)	78 (71–83)	0.341
Male, *n* (%)	15 (71)	4 (50)	0.242
BMI, kg/m^2^	25 (23–27)	26 (24–31)	0.241
STEMI, *n* (%)	11 (52)	5 (63)	0.697
NSTEMI, *n* (%)	10 (48)	3 (37)	0.697
Lesion coronary artery, *n*	2 (1–3)	1 (1-2)	0.189
Three-vessel coronary artery disease, *n* (%)	7 (33)	1 (13)	0.381
Killip class, *n* (%)			0.804
0	2 (10)	1 (13)	
1	17 (85)	7 (88)	
2	1 (5)	0 (0)	
Stent implantation, *n* (%)			
DES	11 (52)	1 (13)	0.093
BMS	9 (43)	8 (100)	0.009
Risk factors, *n* (%)			
Smoking	8 (38)	1 (13)	0.371
Hypertension	13 (62)	5 (63)	1.000
Hyperlipidemia	4 (19)	3 (38)	0.357
Diabetes mellitus	3 (14)	5 (63)	0.019
Family history of CAD	1 (5)	1 (13)	0.483
Medical history, *n* (%)			
PCI	1 (5)	1 (13)	0.483
CABG	0 (0)	1 (13)	0.276
LEAD	2 (10)	0 (0)	1.000
Ictus	0 (0)	0 (0)	—
Renal failure	1 (5)	0 (0)	1.000
Atrial fibrillation	2 (10)	1 (13)	1.000
COPD	2 (10)	1 (13)	1.000
Medical therapy, *n* (%)			
Ca-antagonist	3 (14)	1 (13)	1.000
Ca-channel blockers	0 (0)	0 (0)	—
ACEi	13 (62)	4 (50)	0.683
ARB	2 (10)	1 (13)	1.000
*β*-blockers	10 (48)	5 (63)	0.682
Statins	21 (100)	8 (100)	—
GPIIb/IIIa inhibitors	7 (33)	3 (38)	1.000

Data are expressed as median and interquartile range (25th–75th) or number (percentage).

ACEi: angiotensin-converting enzyme inhibitor; ARB: angiotensin receptor blockers; BMI: body mass index; Ca: calcium; BMS: bare metal coronary stent; CABG: coronary artery by-pass grafting; CAD: coronary artery disease; COPD: chronic obstructive pulmonary disease; DES: drug-eluting stent; LEAD: lower extremity arterial disease; GP: glycoprotein; PCI: percutaneous coronary intervention; NSTEMI; non-ST-segment elevation myocardial infarction.

**Table 2:** Inflammatory pattern at 24 hours from daily administration of dual antiplatelet treatment in clopidogrel responders and nonresponders.

	All cases (*n* = 29)	Responders (*n* = 21)	Nonresponders (*n* = 8)	*P*
Th1/Th2 milieu				
IL-4, pg/mL	0.66 (0.27–1.28)	0.57 (0–0.76)	1.28 (0.84–4.67)	0.003
IL-12, pg/mL	69.1 (44.1–106.2)	59.5 (36.5–101.6)	91.4 (63.0–119.7)	0.106
IFN*γ*, pg/mL	63.2 (49.4–99.1)	55.2 (43.9–88.5)	93.1 (60.2–127.3)	0.066
Proinflammatory cytokines				
IL-6, pg/mL	5.7 (3.2–19.9)	5.1 (3.2–20.0)	9.7 (3.5–1406.4)	0.381
TNF-*α*, pg/mL	2.9 (0.7–10.9)	2.4 (0–5.5)	9.4 (0.8–23.2)	0.283
Anti-inflammatory cytokines				
IL-1ra, pg/mL	750 (595–1222)	713 (563–1093)	1005 (675–2686)	0.218
IL-10, pg/mL	25.4 (13.1–34.5)	20.3 (11.2–28.7)	32.2 (25.7–38.3)	0.079
Chemokines				
I-TAC, pg/mL	165 (69–206)	177 (162–228)	74 (0–170)	0.080
IP10, pg/mL	965 (726–2200)	949 (726–2223)	1532 (698–2118)	0.897
MCP-1, pg/mL	17.3 (14.9–31.8)	17.3 (14.8–31.8)	21.1 (15.0–31.3)	0.775
MIP-1*β*, pg/mL	94.5 (66.7–115.1)	78.1 (58.2–108.4)	115.3 (96.3–131.3)	0.016

Data are expressed as median and interquartile range (25th–75th).

IL: interleukin; IFN: interferon; IP10: interferon-gamma-induced protein 10; I-TAC: interferon-inducible T cell alpha chemoattractant; MCP-1: monocyte chemotactic protein-1; MIP-1*β*: macrophage inflammatory protein-1*β*; TNF: tumor necrosis factor; Th1/Th2: T helper type-1 or type-2 cells.

**Table 3:** Redox status at 24 hours from daily administration of dual antiplatelet treatment between clopidogrel responders and nonresponders.

	All cases (*n* = 23)	Responders (*n* = 17)	Nonresponders (*n* = 6)	*P*
r-Hcy_pl_, *μ*mol/L	0.19 (0.14–0.20)	0.20 (0.15–0.25)	0.17 (0.10–0.19)	0.227
t-Hcy_pl_, *μ*mol/L	13.5 (8.6–15.3)	13.0 (8.6–15.2)	13.9 (9.8–16.6)	0.812
r-GSH_pl_, *μ*mol/L	3.93 (1.64–5.59)	4.25 (1.54–6.24)	3.52 (1.71–4.50)	0.412
t-GSH_pl_, *μ*mol/L	6.10 (2.78–7.08)	6.26 (3.46–7.08)	3.84 (2.60–7.46)	0.560
r-GSH_bl_, *μ*mol/L	426 (393–500)	451 (410–537)	392 (315–431)	0.033
t-GSH_bl_, *μ*mol/L	998 (784–1099)	1031 (876–1108)	873 (716–1054)	0.294
GPx-1, IU/grHb	12.8 (9.3–17.8)	12.6 (9.3–13.4)	19.5 (8.7–27.5)	0.203

Data are expressed as median and interquartile range (25th–75th).

Bl: blood; GSH: glutathione; GPx-1: glutathione peroxidase type-1; GPx-3: glutathione peroxidase type-3; pl: plasma; r: reduced; t: total.

**Table 4:** Time-dependent changes of Hcy and chemotactic molecules levels.

	Responders				Nonresponders			
	24 hours	1 week	1 month	*P*	24 hours	1 week	1 month	*P*
r-Hcy_pl_, *μ*mol/L	0.20 (0.15–0.25)	0.20 (0.14–0.20)	0.20 (0.15–0.30)	0.307	0.17 (0.10–0.19)	0.18 (0.12–0.22)	0.21 (0.12–4.48)	0.050
t-Hcy_pl_, *μ*mol/L	13.0 (8.6–15.2)	12.1 (10.9–14.6)	12.1 (10.4–15.7)	0.936	13.9 (9.8–16.6)	11.6 (9.4–13.2)	12.3 (9.9–36.2)	0.368

I-TAC, pg/mL	177 (162–228)	207 (88–292)	187 (89–268)	0.144	74 (0–170)	76 (0–202)	32 (0–224)	0.223
IP10, pg/mL	949 (726–2223)	986 (817–1317)	1060 (765–1550)	0.344	1532 (698–2118)	1135 (877–2764)	1122 (941–2751)	0.819
MCP-1, pg/mL	17.3 (14.8–31.8)	19.5 (11.0–24.5)	19.5 (15.5–31.1)	0.420	21.1 (15.0–31.3)	41.7 (20.1–50.3)	28.1 (15.4–31.8)	0.819
MIP-1*β*, pg/mL	78.1 (58.2–108.4)	65.7 (50.1–108.7)	75.4 (45.5–94.0)	0.549	115.3 (96.3–131.3)	99.1 (84.8–120.9)	124.6 (72.2–140.8)	0.549

Data are expressed as median and interquartile range (25th–75th).

pl: plasma; r: reduced; t: total; I-TAC: interferon-inducible T cell alpha chemoattractant; MCP-1: monocyte chemotactic protein-1; MIP-1*β*: macrophage inflammatory protein-1*β*; TNF: tumor necrosis factor; Th1/Th2: T helper type 1 or type 2 cells.

*P*: time-dependent changes (24 hours, 1 week, and 1 month) in groups assessed by nonparametric Friedman test.

## Abbreviations

ACS: Acute coronary syndrome
ADP: Adenosine 5′-diphosphate
ASA: Aspirin
CD: Cluster of differentiation
CH_3_CN: Acetonitrile
CXCL: Chemokine (C-X-C motif) ligand
CXCR: Chemokine (C-X-C motif) receptor
GPx-1: GSH peroxidase type-1
Hcy: Homocysteine
HPLC: High-performance liquid chromatography
IFN-*γ*: Interferon-gamma
IL: Interleukin
IL-1ra: Receptor antagonist of IL-1
IP-10, I-TAC: Interferon-inducible T cell alpha chemoattractant
I-TAC: Interferon-gamma-induced protein 10
LC-MSMS: Liquid chromatography-tandem mass spectrometry
MCP-1: Monocyte chemotactic protein-1
MIP-1*β*: Macrophage inflammatory protein-1*β*
NO: Nitric oxide
NR: Nonresponders
PCA: Principal component analysis
PCI: Percutaneous coronary intervention
PRDX4: Peroxiredoxin-4
PRP: Platelet-rich plasma
R: Responders
r-GSH_bl_: Reduced form of blood GSH
ROS: Reactive oxidant species
t-GSH_bl_: Total forms of blood GSH
TNF-*α*: Tumor necrosis factor alpha
TRFE: Transferrin.
